# Host Genetic Determinants of SARS-CoV-2 Susceptibility in Transplant Recipients and Donor Candidates: A Multicohort Analysis of HLA Variation

**DOI:** 10.3390/biomedicines14102352

**Published:** 2026-10-16

**Authors:** Makbule Nihan Somuncu, Mahmut Selman Yıldırım, Ayşe Gül Zamani, Zahide Aleyna Yılık, Sümeyye Sultan Var

**Affiliations:** 1 Tissue Typing Laboratory, Department of Medical Genetics, Faculty of Medicine, Necmettin Erbakan University, Konya 42090, Türkiye; mselman@erbakan.edu.tr (M.S.Y.); agzamani@erbakan.edu.tr (A.G.Z.); 23840601001@erbakan.edu.tr (Z.A.Y.); 2 Tissue Typing Laboratory, Koç University Hospital, İstanbul 34010, Türkiye; 23840601002@erbakan.edu.tr

**Keywords:** COVID-19, genetic susceptibility, HLA, host factors, SARS-CoV-2

## Abstract

**Background:** Host genetic variation plays a key role in susceptibility to SARS-CoV-2 infection. Human leukocyte antigen (HLA) variation influences antiviral immune responses; however, allele-level associations with SARS-CoV-2 infection have varied across populations and clinical groups. This study aimed to evaluate HLA associations with SARS-CoV-2 positivity among transplant recipients and immunocompetent donor candidates representing different clinical and immunological settings. **Methods:** A retrospective multicohort genetic association study was conducted using data from a single-center tissue typing laboratory during the COVID-19 pandemic. The study included *n* = 158 pediatric transplant recipients, n = 159 pediatric donor candidates, *n* = 311 adult transplant recipients, and *n* = 336 adult donor candidates. SARS-CoV-2 infection was defined by documented positive real-time polymerase chain reaction testing. HLA-A, HLA-B, HLA-C, HLA-DRB1, and HLA-DQB1 loci were analyzed using a predefined carrier threshold (≥5). Associations with SARS-CoV-2 positivity were evaluated using Fisher’s exact test with false discovery rate correction and multivariable logistic regression adjusted for age, sex, vaccination status, and antiviral use. **Results:** HLA-DRB1*11 showed a consistent direction of association with higher odds of SARS-CoV-2 positivity across several cohorts, with the strongest statistical support observed in the pediatric transplant and pediatric donor cohorts. In adult solid organ transplant recipients, the association met only the predefined exploratory threshold (q = 0.12). HLA-B*51 and HLA-A*32 were associated with higher odds of positivity in pediatric and adult transplant recipients, respectively, whereas HLA-DRB1*14 was associated with lower odds of positivity in pediatric donor candidates and adult transplant recipients; both HLA-DRB1*14 findings met the predefined exploratory threshold (q < 0.20). Exploratory subgroup analyses indicated that HLA-B*38 was associated with higher vaccination uptake and lower infection frequency (*p* = 0.043); however, these observations were based on small carrier numbers and should be interpreted cautiously. No allele reached the predefined exploratory threshold in healthy adult donor candidates. **Conclusions:** The observed HLA associations differed across the four study cohorts, with several exploratory signals identified among transplant recipients. Because the cohorts also differed in demographic and clinical characteristics, these findings do not establish modification of HLA effects by immune status and require validation in appropriately matched independent populations.

## 1. Introduction

Human leukocyte antigen (HLA) genes encode the major histocompatibility complex (MHC) molecules that present pathogen-derived peptides to T lymphocytes, thereby orchestrating adaptive immune responses. Class I HLA molecules are expressed on nearly all nucleated cells and primarily present intracellularly derived peptides to CD8^+^ T lymphocytes, thereby enabling cytotoxic T-cell responses. In contrast, class II HLA molecules are expressed predominantly on professional antigen-presenting cells and primarily present extracellularly derived peptides to CD4^+^ T lymphocytes, which coordinate and regulate adaptive immune responses [1]. The high degree of polymorphism across classical MHC class I loci (HLA-A, HLA-B, and HLA-C) and MHC class II loci (HLA-DRB1 and HLA-DQB1) contributes to interindividual variation in antigen presentation and adaptive immune responses, making HLA variation a candidate determinant of susceptibility to viral infections [2,3]. Since the emergence of SARS-CoV-2, numerous studies have investigated HLA associations with COVID-19 susceptibility and severity; however, findings have been inconsistent across populations and allele groups. This heterogeneity likely reflects differences in population ancestry and corresponding HLA allele frequencies, linkage disequilibrium structure, cohort composition, definitions of infection and disease severity, SARS-CoV-2 exposure and testing patterns, and statistical approaches [4].

Several large cohort studies have reported associations between HLA allele groups at MHC class I and MHC class II loci and SARS-CoV-2 infection or disease progression [5,6]. In particular, variation at MHC class II loci, including HLA-DRB1, has been investigated in relation to COVID-19 severity and antigen presentation [7]. A population-based study from West Africa reported an association between HLA-DRB1*11 and higher odds of SARS-CoV-2 positivity, particularly among asymptomatic individuals, and identified HLA-DRB1*12 as a secondary allele group of interest [8]. However, large multicohort analyses have not consistently replicated classical HLA associations, highlighting the modest effect sizes and complex genetic architecture underlying COVID-19 susceptibility [9,10].

Despite these studies, few investigations have systematically evaluated HLA associations across cohorts representing contrasting immunological states—such as immunocompromised transplant recipients and immunocompetent donors—using standardized allele-level analytic frameworks [11,12]. This gap is particularly relevant given that transplant recipients are exposed to chronic immunosuppression and exhibit altered T-cell repertoire dynamics, conditions under which HLA-mediated effects on viral susceptibility may differ substantially from those observed in immunologically intact populations.

From a genetic epidemiology perspective, HLA loci represent some of the most polymorphic regions of the human genome and are key determinants of interindividual variability in adaptive immune responses. We hypothesized that patterns of HLA association with SARS-CoV-2 positivity might differ across transplant recipients and immunocompetent donor candidates. We therefore performed cohort-specific allele group-level analyses using a uniform analytical framework. These analyses were intended to compare observed association patterns across cohorts and did not constitute a formal test of effect modification by immune or transplant status.

## 2. Materials and Methods

### 2.1. Study Population and Design

This retrospective multicohort genetic association study used clinical and laboratory data from the institutional tissue typing laboratory and hospital electronic medical records. Records of individuals evaluated between April 2020 and December 2022 were screened for eligibility without sampling on the basis of SARS-CoV-2 PCR outcome. Of 1091 records reviewed, 964 unrelated individuals with the required HLA typing, clinical data, and documented PCR results were included. Participants were assigned to clinical cohorts according to transplant or donor-candidate status and were then classified according to documented SARS-CoV-2 PCR positivity.

Transplant cohorts included individuals with a confirmed history of hematopoietic stem cell transplantation or solid organ transplantation, whereas control cohorts comprised immunocompetent unrelated donor candidates without a history of transplantation or immunodeficiency. These individuals were included solely as unrelated control populations for genetic comparison and were not considered transplant donors within any clinical decision-making framework. To minimize potential bias arising from shared HLA haplotypes, only unrelated individuals with complete data from a single center were included, and analyses were performed separately within each cohort.

Participants were stratified into four cohorts: pediatric hematopoietic stem cell transplant recipients (*n* = 158), healthy pediatric donor candidates (*n* = 159), adult solid organ transplant recipients (*n* = 311), and healthy adult donor candidates (*n* = 336). All 158 pediatric transplant recipients underwent hematopoietic stem cell transplantation using bone marrow as the stem-cell source, whereas all 311 adult solid-organ transplant recipients underwent kidney transplantation.

Because of the retrospective design, all available SARS-CoV-2 RT-PCR results in the institutional medical records were reviewed longitudinally from the beginning of the observation period until either the first documented positive result or the end of the study period. Participants were classified as COVID-19 positive if at least one positive RT-PCR result was documented during this interval. COVID-19 negative status required at least one documented negative RT-PCR result and the absence of any subsequent positive RT-PCR result through the end of the observation period. SARS-CoV-2 testing was performed using the Ds Coronex COVID-19 Multiplex Real-Time qPCR Test Kit (DS Bio and Nano Technologies, Ankara, Türkiye), which targets the viral ORF1ab and N genes and includes human RNase P as an internal control.

Demographic variables, including age and sex, as well as vaccination status, vaccine type and number of doses, antiviral treatment, hospitalization status, hospital length of stay, and infection frequency, were retrieved from institutional electronic medical records.

### 2.2. Inclusion and Exclusion Criteria

Eligible participants had available HLA typing data, complete demographic and clinical information, and documented SARS-CoV-2 PCR results. Individuals with incomplete data, missing PCR results, or biological relatedness to other donor candidates were excluded to reduce potential bias.

### 2.3. HLA Typing

HLA typing data were originally generated in the institutional tissue typing laboratory as part of routine workflows supporting transplant evaluation. The present study represents a retrospective secondary analysis of these pre-existing datasets conducted within a genetic epidemiology framework. The analysis was performed independently of clinical transplant decision-making, donor eligibility assessment, and donor–recipient matching processes and did not influence transplantation practices.

Peripheral blood samples were collected in EDTA tubes during routine clinical care. Genomic DNA was extracted using the QIAamp DNA Mini Kit (Qiagen, Germany) according to the manufacturer’s instructions. Low-resolution typing of classical MHC class I loci (HLA-A, HLA-B, and HLA-C) and MHC class II loci (HLA-DRB1 and HLA-DQB1) was performed using polymerase chain reaction–sequence-specific oligonucleotide (PCR-SSO) methodology. Accordingly, all study-derived HLA assignments and association analyses were reported at the allele group (first-field) level; two-field high-resolution alleles were not resolved. Hybridization and detection were carried out using the Luminex 2.0 platform (Luminex Corporation, Austin, TX, USA). Allele assignments were based on IPD-IMGT/HLA database nomenclature, v3.52, in accordance with World Health Organization HLA nomenclature guidelines. All analyses were performed using anonymized data without access to clinical transplant decision pathways.

### 2.4. Statistical Analysis

All statistical analyses were performed using R statistical software (R Foundation for Statistical Computing, Vienna, Austria), v4.5.3. All statistical tests were two-sided. For regression-based analyses, a *p*-value < 0.05 was considered statistically significant. Analyses were conducted separately within each cohort using a standardized allele group-level analytical framework.

To improve inferential stability, a minimum carrier threshold of *n* ≥ 5 was applied to reduce sparse-cell bias. For allele group-level screening, Benjamini–Hochberg false discovery rate (FDR) correction was applied across all eligible tested allele groups within each cohort. Each cohort constituted a separate testing family because the cohorts represented distinct clinical populations. A q-value < 0.20 was used as the exploratory screening threshold, whereas q < 0.05 indicated stronger statistical support within the cohort-wide FDR framework. This approach does not control the FDR across all four cohorts. Penalized regression was used where model separation occurred, and distribution-specific models were selected for skewed or count outcomes. Each penalized regression was used where model separation occurred, and distribution-specific models were selected for skewed or count outcomes.

#### 2.4.1. Demographic Comparisons

Normality of continuous variables was assessed using the Shapiro–Wilk test. Because age distributions were non-normal across cohorts, continuous variables were compared using the Mann–Whitney U test. Categorical variables were compared using Pearson’s chi-square test or Fisher’s exact test, as appropriate.

#### 2.4.2. HLA Allele Group Association Analysis

HLA alleles at the HLA-A, HLA-B, HLA-C, HLA-DRB1, and HLA-DQB1 loci were analyzed separately within each cohort. A predefined carrier threshold of ≥5 individuals per allele was applied to reduce sparse-cell bias. Associations with SARS-CoV-2 positivity were evaluated using Fisher’s exact test. Odds ratios (ORs) and 95% confidence intervals (CIs) were calculated from 2 × 2 contingency tables. Hardy–Weinberg equilibrium was not formally assessed in the healthy donor cohorts.

To address multiple testing, *p*-values were adjusted using the Benjamini–Hochberg FDR procedure across all eligible tested allele groups within each cohort, as described above. Findings with 0.05 ≤ q < 0.20 were interpreted as exploratory, whereas q < 0.05 indicated stronger statistical support within the cohort-wide FDR framework.

#### 2.4.3. Multivariable Modeling

Multivariable regression constituted a secondary, exploratory stage following the allele group-level screening described above. Only alleles meeting both the predefined carrier threshold and the exploratory cohort-wide FDR threshold for SARS-CoV-2 positivity (q < 0.20) were entered into adjusted models. This two-stage procedure was used to assess whether the direction and magnitude of the screened associations were retained after covariate adjustment, not to provide independent confirmation. Because allele selection and adjusted modeling were based on the same outcome data, the adjusted estimates are subject to post-selection bias and should be interpreted as hypothesis-generating. Logistic regression was used to assess associations with SARS-CoV-2 positivity and hospitalization. Hospitalization analyses were restricted to allele groups selected through the SARS-CoV-2 positivity screening and did not involve independent hospitalization-based screening or validation. These secondary analyses were subject to selection and additional multiplicity and were interpreted as hypothesis-generating. Models were adjusted for age, sex, vaccination status, and vaccine dose number. In transplant recipient cohorts, antiviral use was additionally included as a covariate. Within each cohort, the same covariate specification was applied to all selected allele groups, irrespective of carrier or outcome-event counts. The ≥5-carrier threshold was a screening criterion and did not ensure adequate information for multivariable estimation. Although Firth regression was used where appropriate to address separation and reduce small-sample bias, it could not overcome limited statistical power or imprecision arising from sparse carrier and event counts. Time since transplantation, underlying indication for transplantation, and detailed information on immunosuppressive regimens or treatment intensity were not available in the retrospective dataset and therefore could not be included as covariates.

When small cell counts or quasi-complete separation were present, Firth-penalized logistic regression was applied to obtain bias-reduced estimates. COVID-19 infection frequency was evaluated using negative binomial regression. Accordingly, all adjusted estimates were treated as secondary and exploratory.

## 3. Results

### 3.1. Pediatric Hematopoietic Stem Cell Transplant Recipients (Cohort 1)

A total of 158 pediatric hematopoietic stem cell transplant recipients were analyzed (COVID-19 positive: *n* = 81; COVID-19 negative: *n* = 77). Age distribution was non-normal (Shapiro–Wilk *p* < 0.001), with no significant difference between groups (median 7 years [IQR 2–10] vs. 8.5 years [IQR 4–13]; Mann–Whitney *p* = 0.578). Sex distribution was comparable (χ^2^ *p* = 0.949).

**Figure 1:**
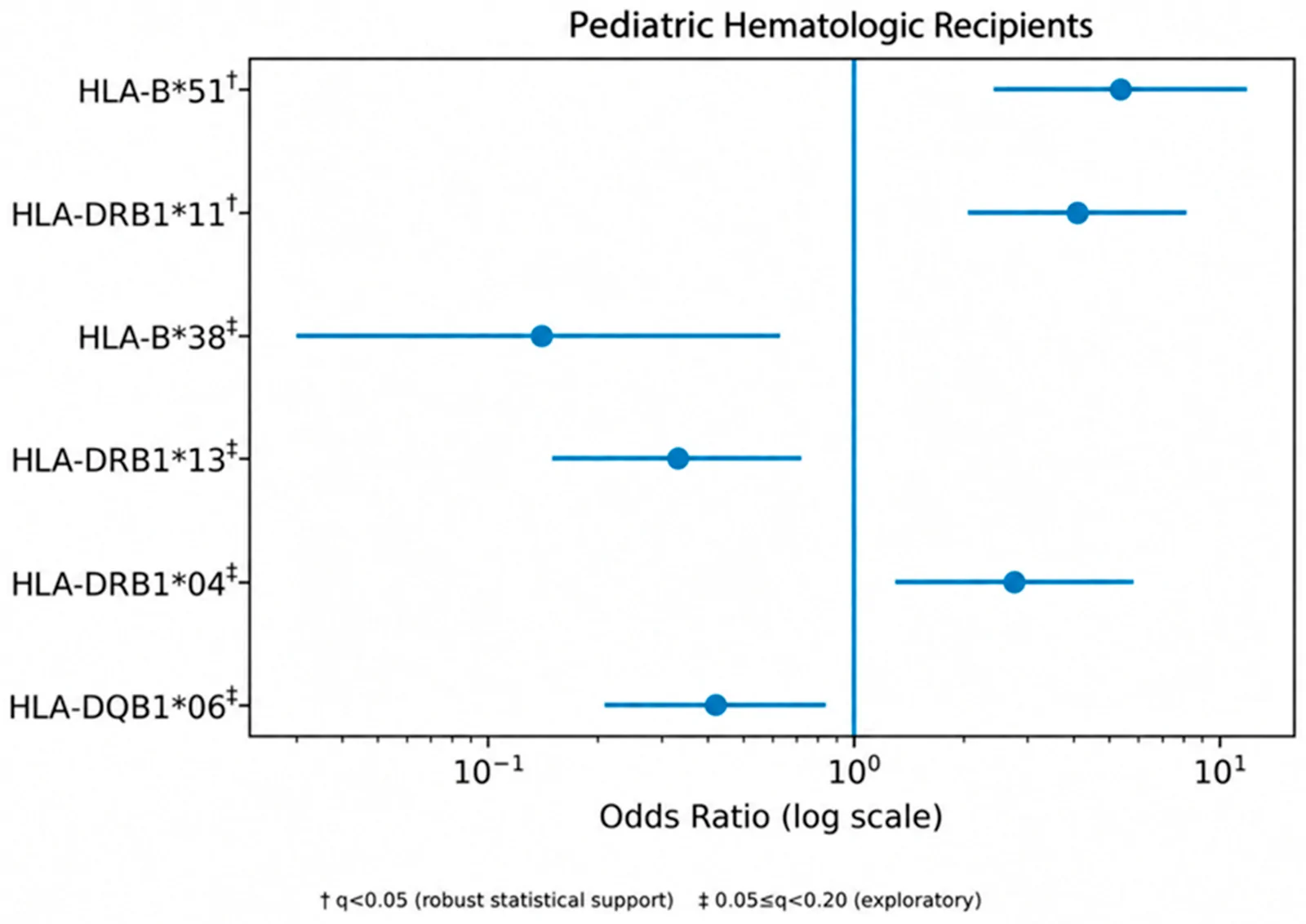
HLA allele associations with SARS-CoV-2 positivity in pediatric hematopoietic stem cell transplant recipients. Forest plot showing adjusted odds ratios (aORs) and 95% confidence intervals (CIs) for the association between selected HLA alleles and SARS-CoV-2 positivity in pediatric hematopoietic stem cell transplant recipients. Alleles were included based on a predefined carrier threshold (≥5 individuals) and exploratory significance following Benjamini–Hochberg false discovery rate (FDR) correction within each cohort (FDR < 0.20). Because allele selection and adjusted modeling used the same outcome data, these adjusted estimates are secondary and exploratory and do not constitute independent confirmation. Multivariable logistic regression models were adjusted for age, sex, vaccination status, and vaccine dose number; antiviral use was additionally included. Circles represent adjusted effect estimates, with horizontal lines indicating 95% confidence intervals. The vertical reference line at an odds ratio of 1 denotes no association. HLA-B*51 and HLA-DRB1*11 were associated with higher odds of SARS-CoV-2 positivity, whereas HLA-B*38, HLA-DRB1*13, and HLA-DQB1*06 were associated with lower odds of positivity. Cohort-level carrier counts (total carriers; SARS-CoV-2-positive carriers) were: HLA-B*51 (46; 36), HLA-B*38 (14; 2), HLA-DRB1*11 (63; 45), HLA-DRB1*13 (36; 11), HLA-DRB1*04 (42; 29), and HLA-DQB1*06 (47; 17).

Alleles with ≥5 carriers were evaluated. Following cohort-wide false discovery rate correction, six alleles met the predefined exploratory threshold (FDR < 0.20). HLA-B*51 and HLA-DRB1*11 were associated with higher odds of SARS-CoV-2 positivity after cohort-wide FDR correction (FDR < 0.05). Additional exploratory associations were observed for HLA-B*38, HLA-DRB1*13, and HLA-DQB1*06, which were associated with lower odds of positivity, whereas HLA-DRB1*04 was associated with higher odds of positivity (Table 1, Figure 1).

Secondary exploratory models suggested higher odds of hospitalization among carriers of HLA-DRB1*11, HLA-DQB1*06, HLA-B*51, and HLA-DRB1*04, although confidence intervals were wide. These allele groups had been selected based on SARS-CoV-2 positivity rather than hospitalization; therefore, the findings are subject to selection and additional multiplicity and do not constitute independently validated hospitalization associations. No allele was associated with hospital length of stay. In a secondary exploratory Firth-adjusted analysis, HLA-B*38 carriage was associated with higher odds of vaccination uptake (aOR 4.03, 95% CI 1.26–12.87). Among vaccinated participants, none of the six HLA-B*38 carriers were COVID-19 positive, compared with 10 of 21 non-carriers. However, this sparse comparison did not reach conventional statistical significance and was accompanied by a very wide confidence interval (*p* = 0.057; Haldane-corrected OR ≈ 0.08, 95% CI 0.004–1.69). COVID-19 infection frequency was also lower among HLA-B*38 carriers (median 0 [IQR 0–0] vs. 0 [IQR 0–1]; *p* = 0.043). Given the small number of carriers and the secondary subgroup nature of these analyses, these observations should be considered exploratory and hypothesis-generating.

### 3.2. Healthy Pediatric Donor Candidates (Cohort 2)

A total of 159 healthy pediatric donor candidates were included (COVID-19 positive: *n* = 68; COVID-19 negative: *n* = 91). Age and sex distributions did not differ significantly between groups.

**Figure 2:**
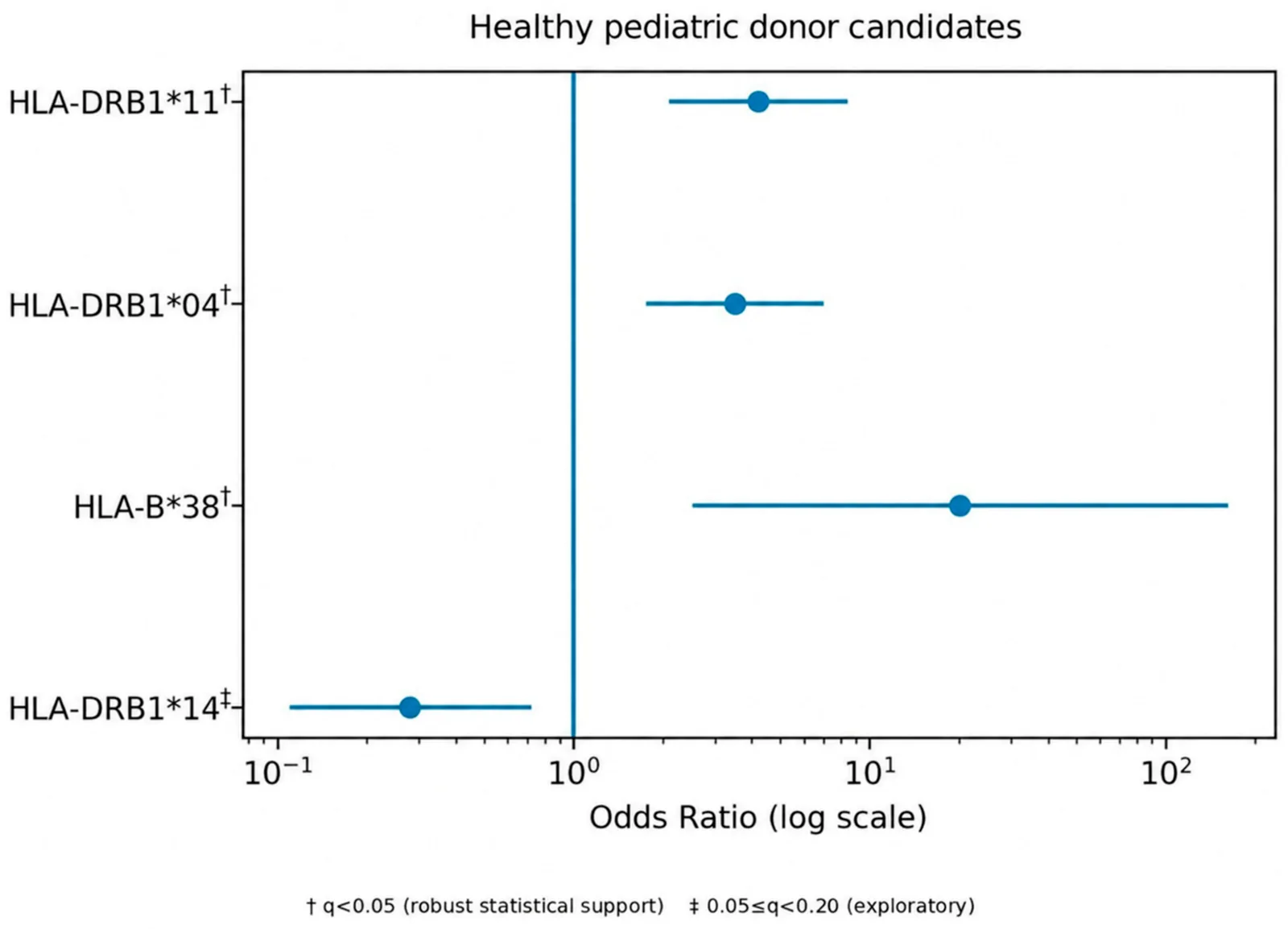
HLA allele associations with SARS-CoV-2 positivity in healthy pediatric donor candidates. Forest plot showing adjusted odds ratios (aORs) and 95% confidence intervals (CIs) for the association between selected HLA alleles and SARS-CoV-2 positivity in healthy pediatric donor candidates. Alleles were included based on a predefined carrier threshold (≥5 individuals) and exploratory significance following Benjamini–Hochberg false discovery rate (FDR) correction within each cohort (FDR < 0.20). Because allele selection and adjusted modeling used the same outcome data, these adjusted estimates are secondary and exploratory and do not constitute independent confirmation. Multivariable logistic regression models were adjusted for age, sex, vaccination status, and vaccine dose number. Circles represent adjusted effect estimates, with horizontal lines indicating 95% confidence intervals. The vertical reference line at an odds ratio of 1 denotes no association. HLA-DRB1*11, HLA-DRB1*04, and HLA-B*38 were associated with higher odds of SARS-CoV-2 positivity, whereas HLA-DRB1*14 was associated with lower odds of positivity. The first three associations met the q < 0.05 threshold, whereas the HLA-DRB1*14 finding met only the predefined exploratory threshold (q = 0.0737). Cohort-level carrier counts (total carriers; SARS-CoV-2-positive carriers) were: HLA-B*38 (11; 10), HLA-DRB1*11 (54; 32), HLA-DRB1*04 (55; 31), and HLA-DRB1*14 (35; 6).

After applying the ≥5-carrier threshold and cohort-wide FDR correction, four alleles met the exploratory threshold (FDR < 0.20). HLA-DRB1*11, HLA-DRB1*04, and HLA-B*38 were associated with higher odds of SARS-CoV-2 positivity, whereas HLA-DRB1*14 was associated with lower odds of positivity. Secondary exploratory multivariable models yielded adjusted estimates in the same direction for these screened alleles (Table 1, Figure 2); these estimates should not be interpreted as independent confirmation. No allele was associated with hospitalization.

### 3.3. Adult Solid Organ Transplant Recipients (Cohort 3)

Among 311 adult solid organ transplant recipients (COVID-19 positive: *n* = 160; COVID-19 negative: *n* = 151), age and sex distributions were comparable.

**Figure 3:**
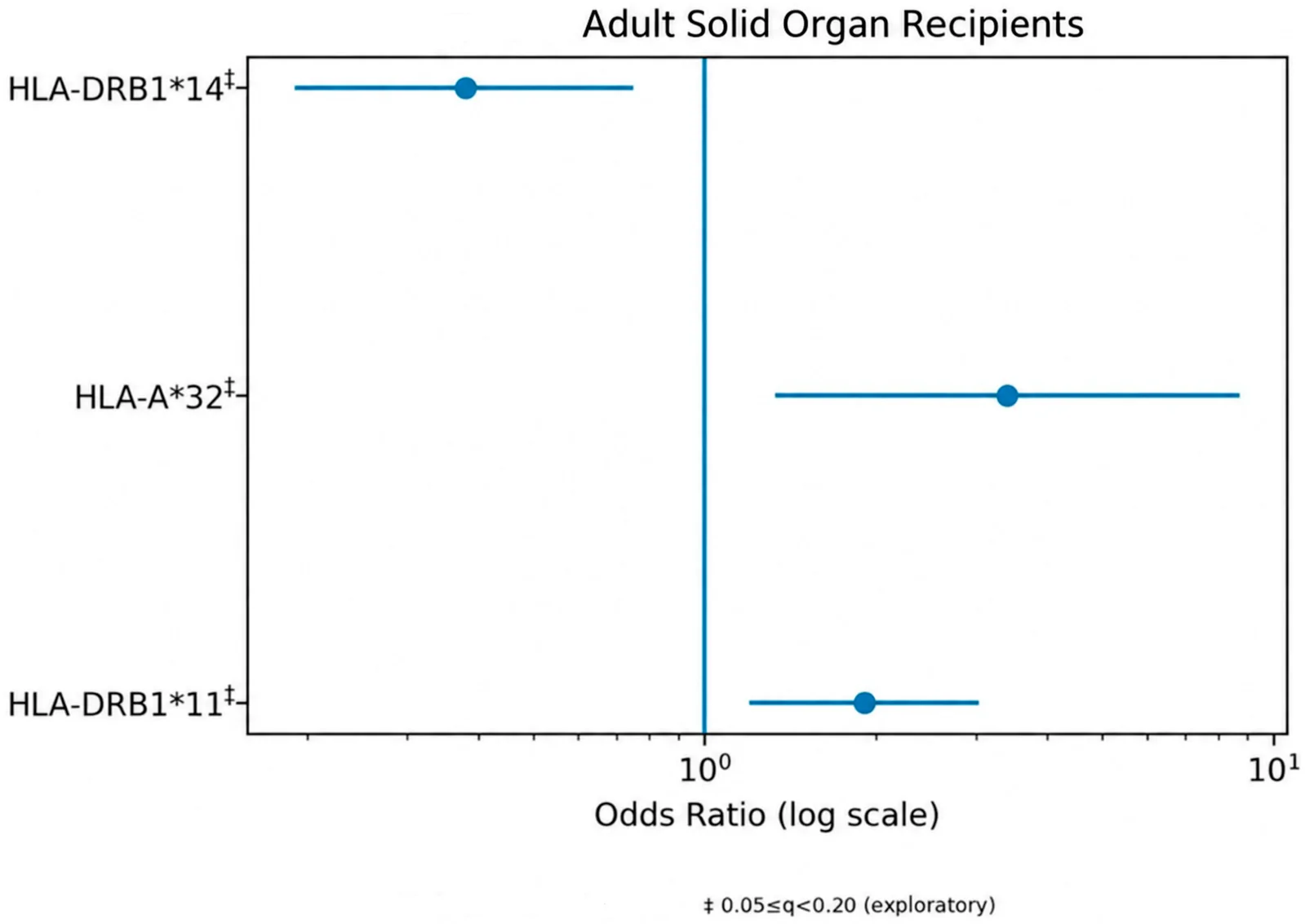
HLA allele associations with SARS-CoV-2 positivity in adult solid organ transplant recipients. Forest plot showing adjusted odds ratios (aORs) and 95% confidence intervals (CIs) for the association between selected HLA alleles and SARS-CoV-2 positivity in adult solid organ transplant recipients. Alleles were included based on a predefined carrier threshold (≥5 individuals) and exploratory significance following Benjamini–Hochberg false discovery rate (FDR) correction within each cohort (FDR < 0.20). Because allele selection and adjusted modeling used the same outcome data, these adjusted estimates are secondary and exploratory and do not constitute independent confirmation. Multivariable logistic regression models were adjusted for age, sex, vaccination status, and vaccine dose number; antiviral use was additionally included. Circles represent adjusted effect estimates, with horizontal lines indicating 95% confidence intervals. The vertical reference line at an odds ratio of 1 denotes no association. HLA-A*32 and HLA-DRB1*11 were associated with higher odds of SARS-CoV-2 positivity, whereas HLA-DRB1*14 was associated with lower odds of positivity; all three findings met only the predefined exploratory threshold (q = 0.12). Cohort-level carrier counts (total carriers; SARS-CoV-2-positive carriers) were: HLA-A*32 (25; 18), HLA-DRB1*11 (121; 69), and HLA-DRB1*14 (44; 14).

Following cohort-wide FDR correction, three alleles met the predefined exploratory threshold (q < 0.20). HLA-DRB1*14 was associated with lower odds of SARS-CoV-2 positivity, whereas HLA-A*32 and HLA-DRB1*11 were associated with higher odds of positivity. All three associations had q = 0.12 and should therefore be interpreted as exploratory. Secondary exploratory multivariable analyses yielded adjusted estimates in the same direction for HLA-DRB1*14, HLA-A*32, and HLA-DRB1*11; however, these estimates should not be interpreted as independent confirmation (Table 1, Figure 3). No allele was associated with hospitalization in the Firth models, and no significant associations were observed for vaccination uptake or antiviral use.

### 3.4. Healthy Adult Donor Candidates (Cohort 4)

A total of 336 healthy adult donor candidates were analyzed. Age and sex distributions were comparable between COVID-19–positive and COVID-19 negative groups.

**Figure 4:**
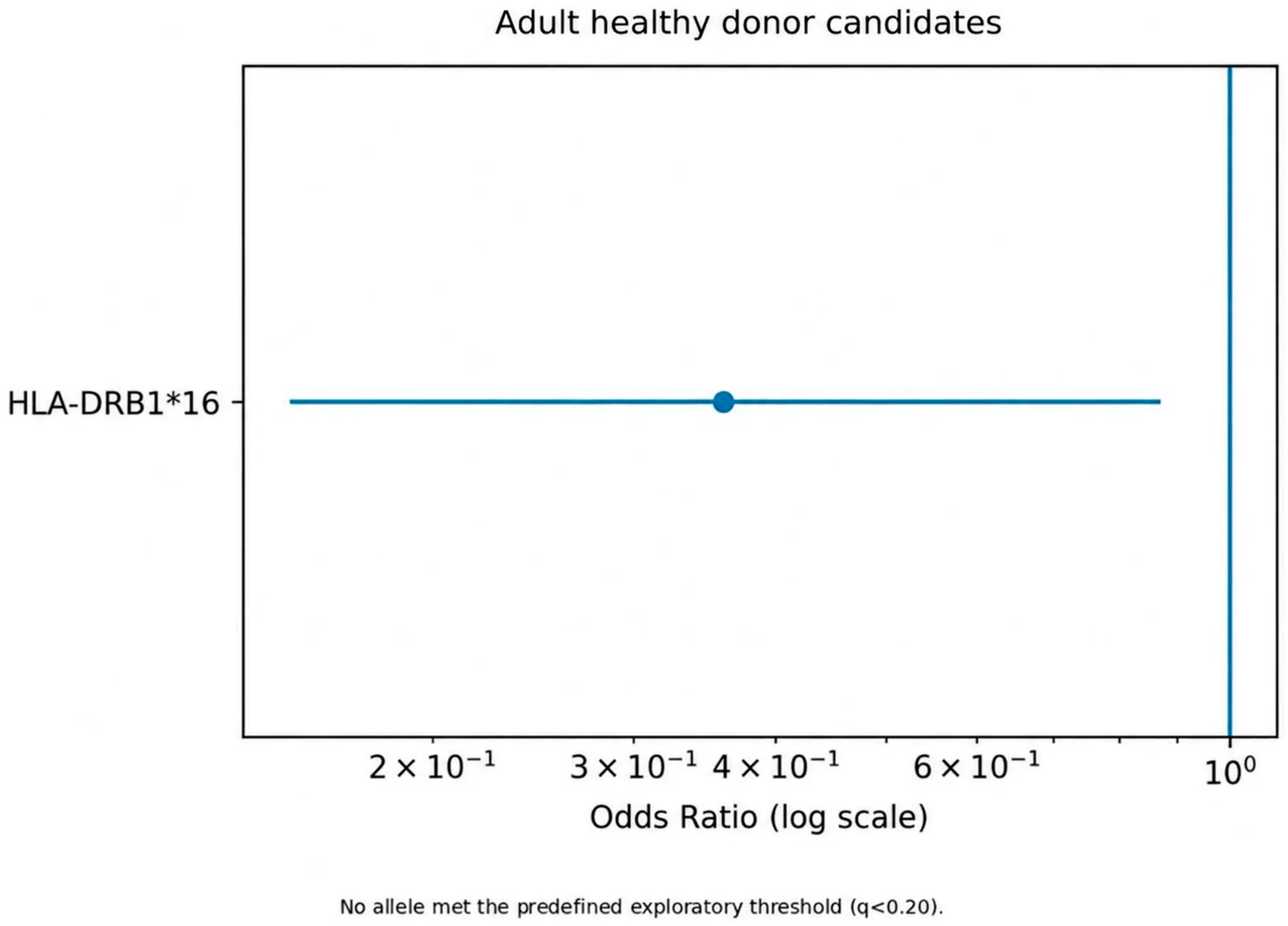
HLA allele associations with SARS-CoV-2 positivity in healthy adult donor candidates. Forest plot showing adjusted odds ratios (aORs) and 95% confidence intervals (CIs) for the association between selected HLA alleles and SARS-CoV-2 positivity in healthy adult donor candidates. Alleles meeting the carrier threshold were screened using cohort-wide Benjamini–Hochberg false discovery rate (FDR) correction. Because no allele met the exploratory threshold (FDR < 0.20), the adjusted estimate for the nominal HLA-DRB1*16 signal is presented only as a secondary exploratory analysis and does not constitute independent confirmation. Multivariable logistic regression models were adjusted for age, sex, vaccination status, and vaccine dose number. Circles represent adjusted effect estimates, with horizontal lines indicating 95% confidence intervals. The vertical reference line at an odds ratio of 1 denotes no association. No HLA allele reached the predefined exploratory significance threshold after FDR correction. A nominal association was observed for HLA-DRB1*16; however, this did not remain significant after adjustment. Cohort-level carrier counts (total carriers; SARS-CoV-2-positive carriers) were: HLA-DRB1*16 (27; 7).

After applying the ≥5-carrier threshold and cohort-wide FDR correction, no allele met the predefined exploratory threshold (FDR < 0.20). A nominal association was observed for HLA-DRB1*16, but did not remain significant after FDR correction (Table 1, Figure 4).

### 3.5. Cross-Cohort Summary

Across the independently analyzed cohorts, HLA-DRB1*11 showed a consistent direction toward higher odds of documented SARS-CoV-2 positivity, with the strongest statistical support observed in pediatric hematopoietic stem cell transplant recipients. HLA-DRB1*14 showed a protective pattern among pediatric donor candidates and adult solid-organ transplant recipients. HLA-B*51 and HLA-A*32 represented cohort-specific risk signals in pediatric and adult transplant recipients, respectively. No allele reached the predefined exploratory FDR threshold in healthy adult donor candidates. These observations reflect qualitative cross-cohort patterns rather than results from a pooled comparison or formal interaction analysis and therefore do not establish an immune-context-dependent effect.

## 4. Discussion

Across the independently analyzed cohorts, HLA-DRB1*11 showed a consistent direction of association with higher odds of SARS-CoV-2 positivity in pediatric hematopoietic stem cell transplant recipients, pediatric donor candidates, and adult solid organ transplant recipients. Statistical support was strongest in the pediatric transplant and donor cohorts, whereas the association in adult solid organ transplant recipients met only the predefined exploratory threshold (q = 0.12), and no corresponding association was observed in healthy adult donor candidates. Taken together, the consistent direction across several cohorts warrants further investigation but does not establish a context-independent or generalizable effect. Replication in independent, ancestry-matched populations is required. In contrast, HLA-DRB1*14 was associated with lower odds of SARS-CoV-2 positivity in more than one cohort. These opposite directions of association warrant investigation with higher-resolution typing and functional assays. Although a formal pooled analysis was not performed, the use of a consistent analytical framework across cohorts enabled direct and interpretable cross-cohort comparison of allele group-level effects. These findings are supported by prior studies demonstrating that HLA variation contributes to SARS-CoV-2 susceptibility and immune heterogeneity at the population level [13]. Allele-specific differences in peptide presentation and T-cell activation have been shown to influence antiviral responses, with variability in CD8^+^ T-cell reactivity linked to HLA genotype [14]. Importantly, emerging evidence suggests that the clinical impact of HLA variation extends beyond infection susceptibility to include modulation of disease severity and immune response dynamics, although effect sizes remain modest and context-dependent [15].

Our cohort-specific analyses identified differences in the pattern and strength of observed HLA associations across transplant recipients and donor candidates. However, these differences cannot be attributed specifically to immune status because the cohorts also differed in age, clinical characteristics, transplant-related factors, healthcare exposure, and potentially SARS-CoV-2 testing patterns. A formal interaction analysis in appropriately matched populations would be required to determine whether immune or transplant status modifies HLA-associated effects. Within the pediatric transplant recipient cohort, HLA-B*38 was associated with increased vaccination uptake (aOR 4.03, 95% CI 1.26–12.87) and demonstrated a trend toward reduced SARS-CoV-2 positivity among vaccinated individuals (0% vs. 47.6%, *p* = 0.057), along with a lower observed infection frequency (*p* = 0.043). However, the vaccinated subgroup included only six HLA-B*38 carriers, and the resulting estimate was imprecise; therefore, this observation should be considered hypothesis-generating and should not be interpreted as evidence of a protective effect or an interaction between HLA-B*38 and vaccination. These cohort-specific secondary analyses identify hypotheses for further investigation but do not establish that HLA variation modifies vaccination response, treatment effects, or clinical course. While previous studies have primarily examined HLA associations in relation to infection susceptibility or disease severity, emerging evidence suggests potential links between host genetic variation and vaccine-induced immune responses, clinical progression, and immune response heterogeneity [16,17]. Notably, although antiviral therapies have been extensively investigated for their clinical impact in COVID-19, their interaction with host HLA genetic variation remains largely unexplored [18,19,20]. While vaccination-related HLA associations have begun to emerge in the recent literature, evidence linking HLA variation to antiviral treatment response remains scarce. In the present study, analyses involving vaccination and antiviral exposure were secondary and exploratory; therefore, they should be interpreted as descriptive, cohort-specific observations requiring independent validation.

Previous studies support a role for HLA-encoded MHC class II molecules in SARS-CoV-2-specific T-cell responses [21,22,23,24,25,26], although reported association patterns vary across populations [27,28]. Because low-resolution typing did not permit reliable allele-specific peptide-binding prediction, the HLA-DRB1*11 and HLA-DRB1*14 associations cannot be linked to specific binding properties or T-cell mechanisms. High-resolution typing, in silico prediction, and peptide-stimulated PBMC studies evaluating CD4^+^ T-cell activation and cytokine responses will be required for mechanistic validation.

Our analysis also identified an exploratory association between the HLA-DQB1*06 allele group and lower odds of SARS-CoV-2 positivity in pediatric hematologic transplant recipients (OR 0.42, 95% CI 0.21–0.84; FDR q = 0.141). At high resolution, HLA-DQB1*06:01 was reported to be more frequent among controls than among hospitalized patients with COVID-19 in one study, suggesting a possible protective association, whereas another study identified HLA-DQB1*06:01 as a risk factor for severe COVID-19 pneumonia [29,30]. Because our low-resolution typing could not distinguish HLA-DQB1*06:01 from other alleles within the HLA-DQB1*06 group, the present allele group-level finding cannot confirm or refute a specific role for HLA-DQB1*06:01.

Large-scale GWAS have consistently shown that host genetic contributions to COVID-19 susceptibility are polygenic and individually modest [31,32]. Against this backdrop, the recurrence of the HLA-DRB1*11 signal in more than one cohort supports further investigation, although the present data cannot determine whether differences in association strength are attributable to immune status or to other demographic and clinical differences between the cohorts.

Class I associations varied across cohorts. HLA-B*51 was associated with higher odds of SARS-CoV-2 positivity in pediatric hematopoietic stem cell transplant recipients. HLA-B*38 showed associations in opposite directions in the two pediatric cohorts. These findings were derived from independently analyzed, clinically distinct populations and should not be regarded as directly comparable replication estimates. The association with lower odds of positivity in pediatric transplant recipients met only the exploratory FDR threshold (q = 0.079), whereas the estimate indicating higher odds in pediatric donor candidates had a wide confidence interval despite meeting the q < 0.05 threshold. Thus, the contrasting directions demonstrate between-cohort heterogeneity but do not establish modification by immune or transplant status. Replication in larger, clinically comparable cohorts is required. Among adult solid organ transplant recipients, HLA-A*32 was associated with higher odds of SARS-CoV-2 positivity; however, this association met only the predefined exploratory threshold (q = 0.12). Previous studies indicate that high-resolution HLA variants encoding MHC class I molecules may differ in peptide presentation and T-cell responses [33,34,35]. However, because the present study used low-resolution typing and did not include functional assays, our allele group-level findings do not permit allele-specific mechanistic inference.

Importantly, external evidence from an independent Turkish kidney transplant cohort further contextualizes these findings. In that study, HLA-B*38 and HLA-DRB1*11 were enriched among patients with severe COVID-19 [36]. The HLA-DRB1*11 finding is directionally consistent with our association with SARS-CoV-2 positivity, whereas the HLA-B*38 result concerns a different outcome and cannot be interpreted as replication of our finding. In addition to the recurrent signals emphasized above, several secondary exploratory associations were observed among pediatric hematopoietic stem cell transplant recipients, including HLA-DRB1*04, HLA-DRB1*13, and HLA-DQB1*06. Because these findings were cohort-restricted and were not replicated in the other study cohorts, they were interpreted cautiously and not prioritized as principal multicohort signals. We therefore focused the discussion on alleles showing either cross-cohort consistency or clinically informative subgroup associations.

Notably, no allele reached the predefined exploratory threshold in healthy adult donor candidates, although this absence of findings may reflect limited statistical power, allele-frequency differences, or other cohort-specific characteristics and should not be interpreted as evidence of attenuated HLA effects in immunocompetent individuals. Beyond the alleles identified in our cohorts, HLA-B*15:01 has been associated with asymptomatic SARS-CoV-2 infection, whereas HLA-DRB1*03 has been associated with a reduced risk of fatal outcome in one population; both outcomes differ from the SARS-CoV-2 positivity evaluated here [17,37]. In an Aegean Turkish cohort, the HLA-DRB1*03 allele group frequency was 9.57% [38]. Among donors in Germany with Turkish parentage, the HLA-B*15:01 allele frequency was 2.07% [39]. These estimates come from different donor populations and should not be assumed to represent our study cohorts. Because our low-resolution typing did not resolve HLA-B*15:01, we could not evaluate this specific allele. Population differences in HLA frequencies may also complicate replication [40].

Integrating high-resolution HLA typing and functional immunological assays will be essential to clarify underlying mechanisms. At present, these exploratory findings do not support clinical risk stratification or individualized management based on HLA genotype. Any potential clinical utility should be considered hypothesis-generating and would require independent replication in larger, ancestry-matched populations, high-resolution HLA typing, and demonstration of incremental predictive value beyond established demographic and clinical risk factors.

## 5. Conclusions

In this retrospective multicohort analysis, several HLA allele groups were associated with SARS-CoV-2 positivity within individual cohorts, although most findings met an exploratory FDR threshold. The pattern of observed associations differed among transplant recipients and donor candidates; however, the present cohort-specific analyses do not demonstrate that immune status modifies or amplifies HLA associations. These hypothesis-generating findings require replication in larger, ancestry-matched and clinically comparable cohorts using high-resolution HLA typing and formal interaction analyses. Accordingly, no clinical utility can be inferred from the present findings. Before HLA profiling can be considered for clinical risk prediction, these associations must be independently replicated using high-resolution typing and shown to provide incremental predictive value beyond established clinical risk factors.

This study represents a retrospective secondary analysis of routinely generated clinical and HLA typing data. No study-wide FDR sensitivity analysis was performed; therefore, the robustness of the principal signals to correction across all four cohorts remains unassessed. Findings with q < 0.05 indicate statistical support within the corresponding cohort-wide testing family and should not be interpreted as study-wide statistical significance. As such, the design is inherently subject to limitations related to non-randomized observational datasets, including potential residual confounding and selection bias. Because the HLA data were originally generated for clinical transplant evaluation rather than for hypothesis-driven research, certain potentially relevant immunological or functional variables were not available for analysis. This retrospective multicohort genetic association study may be subject to selection bias and does not permit causal inference. Because HLA typing was performed at low resolution, the observed allele group associations could not be attributed to specific high-resolution alleles, and allele-specific peptide-binding or functional mechanisms could not be evaluated. Because this study was a retrospective secondary analysis of routine low-resolution HLA typing data and the associations were evaluated using allele group carrier status, Hardy–Weinberg equilibrium testing was not prespecified. Consequently, potential deviations from expected genotype distributions could not be evaluated, which represents a methodological limitation. Nevertheless, the use of a uniform typing methodology across cohorts supported internal consistency. A predefined carrier threshold (≥5 individuals) was applied to reduce sparse-cell instability, and Benjamini–Hochberg correction was performed across all eligible tested allele groups within each cohort. Consequently, the reported q-values do not control the false discovery rate across all HLA comparisons in the study; residual false-positive findings and limited power for rare alleles cannot be excluded. Several secondary subgroup analyses also involved very small numbers, particularly the vaccinated HLA-B*38 subgroup, which included only six carriers. Consequently, these analyses had limited statistical power and produced imprecise effect estimates; their findings should be considered hypothesis-generating and require validation in larger cohorts.

Despite multivariable adjustment for age, sex, vaccination status (including dose number), and antiviral treatment, residual confounding remains possible. Although transplant type was homogeneous within each cohort—all pediatric transplant recipients underwent bone marrow-derived hematopoietic stem cell transplantation and all adult solid-organ transplant recipients underwent kidney transplantation—data on time since transplantation, underlying indication, and immunosuppressive regimen or treatment intensity were unavailable and could not be included in the adjusted analyses. Because these factors may affect SARS-CoV-2 exposure, susceptibility, and clinical outcomes, their omission represents an important limitation. In addition, the cohorts differed in age distribution, underlying diseases, medication exposure, healthcare contact, and the likelihood and frequency of SARS-CoV-2 testing. Because these characteristics were not uniformly available or directly comparable across cohorts, they may partly explain the observed differences in association patterns. Accordingly, the cross-cohort findings should be interpreted as qualitative observations rather than evidence of effect modification by immune status. Although the direction of some signals was similar across cohorts, these observations require confirmation in clinically comparable, ancestry-matched populations with detailed transplant-related data. Individual-level ancestry data were not available, and population stratification could not be assessed or adjusted for. Differences in ancestry composition may therefore have confounded the observed HLA associations, particularly the interpretation of recurrent signals across cohorts and comparisons with external populations. The study period extended from April 2020 to December 2022, during which circulating variants, vaccination availability, testing practices, antiviral use, and public-health measures changed. Calendar period and individual observation time were not included as covariates; differences in these factors between participants or cohorts may therefore have confounded associations with documented PCR positivity. Although all available institutional RT-PCR records were reviewed longitudinally through the end of the observation period, testing was performed as part of routine clinical care rather than according to a fixed prospective testing schedule. Therefore, asymptomatic or unrecognized infections and infections diagnosed outside the institution may not have been captured. A participant with only an early negative PCR test could therefore have had a later undocumented infection and remained classified as negative. Differences in testing frequency and observation duration may have contributed to differential ascertainment of documented positivity. Consequently, some degree of infection-status misclassification cannot be completely excluded and may have influenced the estimated HLA associations. Some allele-specific adjusted models and secondary subgroup analyses were limited by small carrier numbers and sparse outcome cells relative to the number of covariates. Firth penalization could not eliminate this limitation, and estimates with wide confidence intervals should be interpreted cautiously. Model-specific carrier and event counts were not reported, limiting readers’ assessment of model stability. In addition, no formal HLA-by-vaccination or HLA-by-treatment interaction analyses were performed. These subgroup findings should therefore be regarded as exploratory and hypothesis-generating.

The present findings should be interpreted within a genetic epidemiology framework. Given the observational design and allele group-level resolution, functional validation studies are required to confirm the biological relevance of these associations. Future investigations should incorporate high-resolution HLA typing and functional immunological assays to further elucidate the biological mechanisms underlying these associations.

## Figures and Tables

**Table 1:** Associations of HLA allele groups at MHC class I and MHC class II loci with SARS-CoV-2 positivity across transplant and donor candidate cohorts.

Cohort (Total *N*; COVID+/−)	Allele	OR (95% CI)	*p*-Value	FDR (q-Value)	Direction of Association
Pediatric Hematologic Transplant Recipients (*N* = 158; 81/77)	HLA-B*51	5.36 (2.4211.88)	<0.001	0.00103	Higher odds of SARS-CoV-2 positivity
	HLA-DRB1*11	4.10 (2.06–8.14)	<0.001	0.00121	Higher odds of SARS-CoV-2 positivity
	HLA-B*38	0.14 (0.03–0.63)	0.004	0.079	Lower odds of SARS-CoV-2 positivity
	HLA-DRB1*13	0.33 (0.15–0.72)	0.007	0.101	Lower odds of SARS-CoV-2 positivity
	HLA-DRB1*04	2.75 (1.30–5.81)	0.011	0.123	Higher odds of SARS-CoV-2 positivity
	HLA-DQB1*06	0.42 (0.21–0.84)	0.015	0.141	Lower odds of SARS-CoV-2 positivity
Healthy Pediatric Donor Candidates (*N* = 159; 68/91)	HLA-DRB1*11	4.20 (2.09–8.44)	<0.001	0.0029	Higher odds of SARS-CoV-2 positivity
	HLA-DRB1*04	3.51 (1.76–6.97)	<0.001	0.0086	Higher odds of SARS-CoV-2 positivity
	HLA-B*38	20.2 (2.51–162.36)	<0.001	0.006	Higher odds of SARS-CoV-2 positivity
	HLA-DRB1*14	0.28 (0.11–0.72)	<0.001	0.0737	Lower odds of SARS-CoV-2 positivity
Adult Solid Organ Transplant Recipients (*N* = 311; 160/151)	HLA-DRB1*14	0.38 (0.19–0.75)	0.005	0.12	Lower odds of SARS-CoV-2 positivity
	HLA-A*32	3.40 (1.33–8.72)	0.007	0.12	Higher odds of SARS-CoV-2 positivity
	HLA-DRB1*11	1.91 (1.20–3.03)	0.008	0.12	Higher odds of SARS-CoV-2 positivity
Healthy Adult Donor Candidates (*N* = 336; 160/176)	HLA-DRB1*16	0.36 (0.15–0.87)	0.026	0.81	Not significant after FDR correction

HLA allele groups at MHC class I loci (HLA-A, HLA-B, and HLA-C) and MHC class II loci (HLA-DRB1 and HLA-DQB1) with ≥5 carriers were analyzed separately within each cohort. Associations with SARS-CoV-2 positivity were evaluated using Fisher’s exact test based on 2 × 2 contingency tables. Crude odds ratios (OR) and 95% confidence intervals (CI) were calculated accordingly. Benjamini–Hochberg FDR correction was applied across all eligible tested allele groups within each cohort. A q-value < 0.20 was defined as the exploratory screening threshold, whereas q < 0.05 indicated stronger statistical support within the cohort-wide FDR framework. The direction of association reflects the crude OR: OR > 1 indicates higher odds of SARS-CoV-2 positivity, whereas OR < 1 indicates lower odds of positivity.

## Data Availability

The data supporting the findings of this study are available from the corresponding author upon reasonable request. The data are not publicly available due to ethical restrictions and the sensitive nature of the clinical and genetic information.

## Abbreviations

The following abbreviations are used in this manuscript:

aOR	Adjusted odds ratio
CD4	Cluster of differentiation 4
CD8	Cluster of differentiation 8
CI	Confidence interval
COVID-19	Coronavirus disease 2019
DNA	Deoxyribonucleic acid
EDTA	Ethylenediaminetetraacetic acid
FDR	False discovery rate
GWAS	Genome-wide association study
HLA	Human leukocyte antigen
IPD-IMGT/HLA	Immuno Polymorphism Database–International ImMunoGeneTics/Human Leukocyte Antigen Database
IQR	Interquartile range
MHC	Major histocompatibility complex
OR	Odds ratio
PCR	Polymerase chain reaction
PCR-SSO	Polymerase chain reaction–sequence-specific oligonucleotide
SARS-CoV-2	Severe acute respiratory syndrome coronavirus 2
WHO	World Health Organization
